# A Case of Superfætation—Placenta Præira

**Published:** 1861-07

**Authors:** J. H. Apperson

**Affiliations:** Bourbon, Douglas Co., Ill.


ARTICLE V.

### CASE OF SUPERFÆTATION.—PLACENTA PRÆVIA.

EDITORS EXAMINER:—We have at present a case under treatment, of what we call *Superfœtation*, existing for some two years or more, and since the commencement of which, the female has conceived and brought forth a healthy child, at full term, and now six months old. The sac finally pointed externally, and was opened; but the point of interest is, that a large quantity of hair has escaped, varying in length, from two to ten or twelve inches. Please account for the length of the hair. I have it for inspection.

I have had also a case of *Placenta Prævia*, where the fœtus was completely enveloped in the placenta, as in a shut sack; where ergot of known strength had no effect, and upon attempting to introduce the hand, for the purpose of turning it was found impossible to penetrate the placental mass. As a last resort, the placenta was ruptured over the vertex—that being the presenting part—and the child delivered with the forceps. The child was of course lost from previous hemorrhage, but the mother recovered without a bad symptom. I should state that after the child was delivered, I had to introduce the hand and carefully detach the adherent placenta.

Yours respectfully,

J. H. APPERSON.

BOURBON, DOUGLAS CO., ILL., April, 1861.

